# Involvement of intracellular free Ca^2+ ^in enhanced release of herpes simplex virus by hydrogen peroxide

**DOI:** 10.1186/1743-422X-3-62

**Published:** 2006-08-31

**Authors:** Emiko Arimoto, Soichi Iwai, Tetsuro Sumi, Yuzo Ogawa, Yoshiaki Yura

**Affiliations:** 1 Department of Oral and Maxillofacial Surgery II, Osaka University Graduate School of Dentistry, Osaka, Japan; 2 Department of Pathology, Osaka University Graduate School of Dentistry, Osaka, Japan

## Abstract

**Background:**

It was reported that elevation of the intracellular concentration of free Ca^2+ ^([Ca^2+^]i) by a calcium ionophore increased the release of herpes simplex virus type 1 (HSV-1). Freely diffusible hydrogen peroxide (H_2_O_2_) is implied to alter Ca^2+ ^homeostasis, which further enhances abnormal cellular activity, causing changes in signal transduction, and cellular dysfunction. Whether H_2_O_2 _could affect [Ca^2+^]i in HSV-1-infected cells had not been investigated.

**Results:**

H_2_O_2 _treatment increased the amount of cell-free virus and decreased the proportion of viable cells. After the treatment, an elevation in [Ca^2+^]i was observed and the increase in [Ca^2+^]i was suppressed when intracellular and cytosolic Ca^2+ ^were buffered by Ca^2+ ^chelators. In the presence of Ca^2+ ^chelators, H_2_O_2_-mediated increases of cell-free virus and cell death were also diminished. Electron microscopic analysis revealed enlarged cell junctions and a focal disintegration of the plasma membrane in H_2_O_2_-treated cells.

**Conclusion:**

These results indicate that H_2_O_2 _can elevate [Ca^2+^]i and induces non-apoptotic cell death with membrane lesions, which is responsible for the increased release of HSV-1 from epithelial cells.

## Background

Polymorphonuclear leukocytes (PMNs) have been detected in the early cellular infiltrate at sites of herpes simplex virus (HSV) infection [1]. It was also reported that large numbers of PMNs infiltrated the mouse vaginal mucosa within 24 h of the inoculation of HSV type 2 [2]. Activated inflammatory cells are a major source of oxidative stress in inflammatory diseases and during secondary inflammation after an initial toxic insult [3,4]. Exogenous oxygen radicals can be also brought to the oral cavity, the target of HSV type 1 (HSV-1) infection, for therapeutic purpose [5-7]. These findings suggest that HSV-infected epithelial cells can be exposed to oxygen radicals during the infection cycle of HSV.

Freely diffusible hydrogen peroxide (H_2_O_2_) as an oxygen radical can damage DNA directly by penetrating the cell nucleus or indirectly by increasing the intracellular concentration of free Ca^2+ ^([Ca^2+^]i). The peroxidation of membrane phospholipids leads to alterations in Ca^2+ ^homeostasis, which further enhances abnormal cellular activity, causing changes in signal transduction, and cellular dysfunction [8-12]. H_2_O_2 _was cytotoxic to renal tubular epithelial cells and caused a sustained and uncontrolled rise in [Ca^2+^]i that preceded substantial cell injury or irreversible cell death [8].

With regard to viral infection and [Ca^2+^]i, many animal viruses such as cytomegalovirus, poliovirus, coxsackie B3 virus, vaccinia virus, measles virus and rotavirus are known to alter Ca^2+ ^homeostasis as a result of viral gene expression [13-18]. [Ca^2+^]i is elevated after the binding of HSV-1 to its cellular receptor [19]. In the previous study, we found that a calcium ionophore, ionomycin, induced Ca^2+^-dependent cell death and increased the virus release from infected epithelial cells [20]. This suggests that Ca^2+ ^may be the stimulator of viral release. However, what causes the elevation of [Ca^2+^]i *in vivo *has not been clarified. In the present study, we examined the possibility that H_2_O_2 _could affect [Ca^2+^]i in HSV-1-infected epithelial cells. The results suggest that H_2_O_2 _is the candidate to promote the release of HSV-1 at the site of viral infection in a [Ca^2+^]i-dependent manner.

## Results

### Effect of H_2_O_2 _on the amounts of cell-free and cell-associated virus

**Figure 1:**
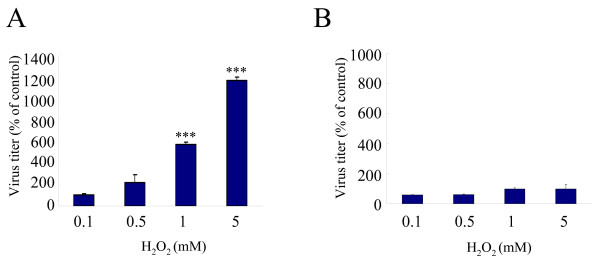
**Effect of H_2_O_2 _on the amount of cell-free virus and cell-associated virus**. FI cells were infected with HSV-1 at an MOI of 2 PFU/cells and cultured for 18 h. Thereafter, cells were treated with H_2_O_2 _at concentrations of 0.1, 0.5, 1 and 5 mM for 2 h, and the amounts of cell-free virus (A) and cell- associated virus (B) in the cultures were determined by plaque assay. Results were compared to those for the controls and a percentage was calculated. Data are means ± SD of three determinations. Differences of means were analyzed with the unpaired *t*-test. * *P *< 0.05, ** *P *< 0.01 and ** *P *< 0.001 vs. samples exposed to H_2_O_2 _only.

In the previous study, we treated HSV-1-infected cells with a calcium ionophore, ionomycin, 18 h post infection (p.i.) in order to detect its enhancing effect on the release of HSV-1[20]. In this condition, most cells attached to the plate and were releasing progeny viruses into culture medium, although further incubation gradually increased the number of detached cells. In the similar condition, we examined the effect of H_2_O_2 _on the release of HSV-1. When FI cells were infected with HSV-1 at a multiplicity of infection (MOI) of 2 plaque forming units (PFU)/cell, cultured for 18 h and treated with H_2_O_2 _at concentrations ranging from 0.1 to 5 mM for 2 h, cell-free virus was increased at 0.5, 1 and 5 mM; the increase at 1 and 5 mM was significant as compared with the untreated control (Fig. 1A). In contrast, the amount of cell-associated virus was not significantly changed (Fig. 1B). In the absence of H_2_O_2_, mean virus titers in cell-free and cell-associated fractions were 4.6 × 10^6 ^and 1.1 × 10^8 ^PFU/ml. After treatment with 1 mM H_2_O_2 _for 2 h, mean virus titers in these fractions were 2.6 × 10^7 ^and 1.1 × 10^8 ^PFU/ml, respectively. A six-fold increase as compared with the untreated control was observed in the cell-free fraction, but no increase was observed in the cell-associated fraction. The proportions of cell-free virus in the total amount of virus in the presence or absence of H_2_O_2 _were 22% and 4%, respectively, indicating that H_2_O_2 _markedly increased cell-free virus in the cultures.

### Effect of H_2_O_2 _on [Ca^2+^] i in HSV-1-infected cells

**Figure 2:**
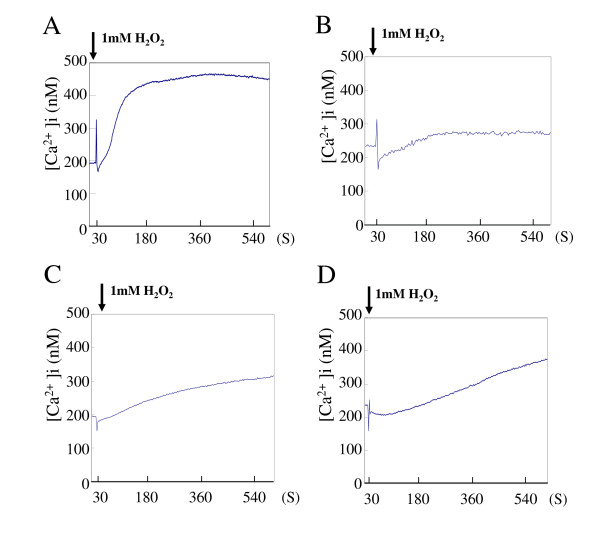
**Effect of H_2_O_2 _on [Ca^2+^]i in HSV-1-infected cells**. HSV-1-infected FI cells were cultured for 18 h. Thereafter, the medium was replaced with Hank's solution and the [Ca^2+^]i was monitored during treatment with 1 mM H_2_O_2 _(A). Alternatively, infected cells were treated with 10 mM EGTA (B), 50 μM BAPTA (C) or 50 μM quin-2 (D) for 20 min prior to treatment with 1 mM H_2_O_2_. Results are representative of 7 independent experiments.

It has been shown that H_2_O_2 _caused a sustained and uncontrolled rise in [Ca^2+^]i that preceded substantial cell injury or irreversible cell death [8]. Whether H_2_O_2 _could affect the [Ca^2+^]i was examined at concentrations to enhance the virus release. FI cells were infected with HSV-1 at an MOI of 2 PFU/cell and cultured for 18 h. The mean level of [Ca^2+^]i in HSV-1-infected cells was approximately 200 nM. When the infected cells were treated with 1 mM H_2_O_2_, a significant rise in [Ca^2+^]i beginning approximately 30 sec after the exposure to H_2_O_2 _was observed. Subsequently, there was a secondary rise in [Ca^2+^]i, that appeared within 40 sec; a maximal level (460 nM) was attained in 6 min (Fig. 2A).

To determine the effect of calcium chelators, infected cells were treated with an extracellular calcium chelating agent, glycol-bis (beta-aminoethyl ether)-N',N',N',N'-tetraacetic acid (EGTA), for 20 min until 18 h p.i., and then H_2_O_2 _treatment was initiated. EGTA did not inhibit the immediate rise in [Ca^2+^]i significantly, but suppressed the secondary rise at a low level (Fig. 2B). When HSV-1-infected cells were exposed to an intercellular Ca^2+ ^chelator, 1,2-bis (2-aminophenoxy)ethane- N',N',N',N'-tetraacetic acid (BAPTA) or quin-2, for 20 min prior to the H_2_O_2 _treatment, both the initial and secondary rises in [Ca^2+^]i were suppressed. Although the secondary rise was suppressed by this treatment, the level of [Ca^2+^]i gradually increased to 300–350 nM in 8 min (Fig. 2C and 2D).

### Effect of buffering [Ca^2+^]i on H_2_O_2_-mediated enhancement of viral release

**Figure 3:**
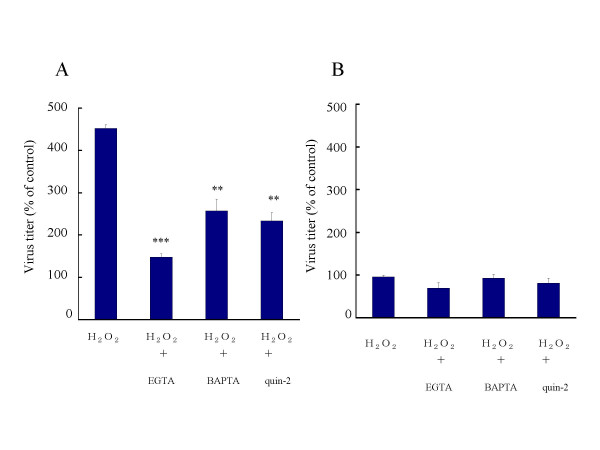
**Effects of Ca^2+ ^depletion on viral release**. HSV-1-infected FI cells were treated with 1 mM H_2_O_2 _from 18 to 20 h p.i. Alternatively, infected cells were pretreated with 10 mM EGTA, 50 μM BAPTA or 50 μM quin-2 for 20 min prior to H_2_O_2 _treatment for 2 h. After treatment with H_2_O_2_, the amounts of cell-free virus (A) and cell- associated virus (B) were determined. Results were compared to those for the controls and a percentage was calculated. Data are means ± SD of three determinations. Differences of means were analyzed with the unpaired *t*-test. * *P *< 0.05, ** *P *< 0.01 and ** *P *< 0.001 vs. samples exposed to H_2_O_2 _only.

The effect of Ca^2+ ^depletion on the release of HSV-1 was examined. Eighteen hours after infection, cells were pretreated with 10 mM EGTA for 20 min to deplete extracellular Ca^2+^. Thereafter, treatment with 1 mM H_2_O_2 _for 2 h was initiated. In this condition, the amount of cell-free virus was 150% of that in the untreated control, whereas it was increased to 450% of the control value by the treatment with H_2_O_2 _(Fig. 3A). The amounts of cell-free virus in the presence of 50 μM BAPTA and 50 μM quin-2 were 250 % and 230 % of the control, respectively, indicating that the H_2_O_2_-mediated increase was diminished by BAPTA and quin-2. The amount of cell-associated virus in the cultures was not significantly altered by H_2_O_2 _in combination with EGTA, BAPTA or quin-2 (Fig. 3B). When HSV-1-infected cells were treated with EGTA, BAPTA or quin-2 only, the amount of cell-free virus was unchanged as compared with that in the untreated control (data not shown).

### Effect of H_2_O_2 _and buffering [Ca^2+^]i on cell viability

**Figure 4:**
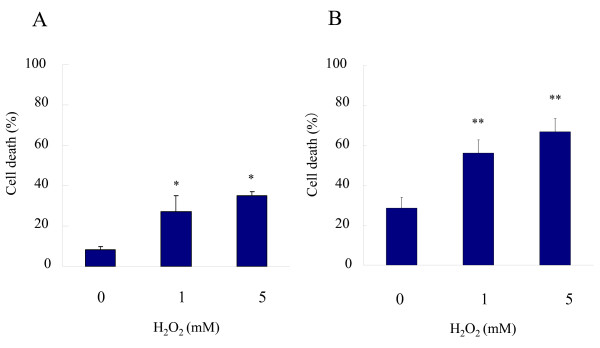
**Effect of H_2_O_2 _on cell viability**. FI cells were treated with 1 mM H_2_O_2 _and stained with trypan blue (A). HSV-1-infected FI cells were treated with 1 mM H_2_O_2 _from 18 to 20 h p.i. and then trypan blue-positive cells were determined (B). Data are means ± SD of three determinations. Differences of means were analyzed with the unpaired *t*-test. * *P *< 0.05 and ** *P *< 0.01 vs. samples exposed to H_2_O_2 _only.

The effect of H_2_O_2 _on cell viability was examined by trypan blue exclusion. In mock-infected FI cells, the proportion of trypan blue-positive dead cells was 8%. After treatment with 1 mM H_2_O_2 _for 2 h, 28% of cells were positive trypan blue (Fig. 4A). When cells were infected with HSV-1 at an MOI of 2 PFU/cell and cultured for 20 h, 29% of cells were stained. After the treatment with 1 mM H_2_O_2 _from 18 to 20 h p.i., the proportion of dead cells was increased to 56% (Fig. 4B). The only detectable morphological change of H_2_O_2_-treated cells was enlargement of intercellular space due to cell rounding, irrespective of HSV-1 infection.

**Figure 5:**
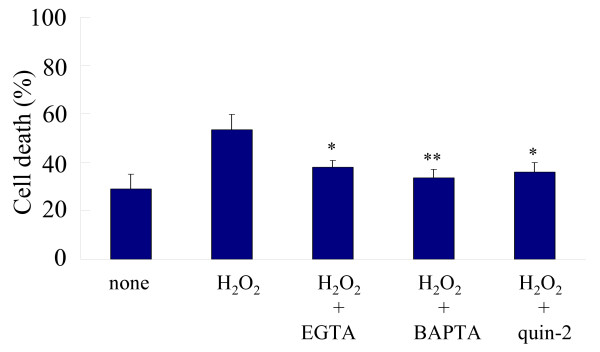
**Effect of Ca^2+ ^depletion on cell viability**. HSV-1-infected FI cells were treated with 1 mM H_2_O_2 _from 18 to 20 h p.i. and then trypan blue-positive cells were determined. For the depletion of extracellular Ca^2+ ^or [Ca^2+^]i, infected cells were pretreated with 10 mM EGTA, 50 μM BAPTA or 50 μM quin-2 for 20 min. Differences of means were analyzed with the unpaired *t*-test. * *P *< 0.05 and ** *P *< 0.01 vs. samples exposed to H_2_O_2 _only.

To determine the effect of Ca^2+ ^chelators, HSV-1-infected cells were pretreated with 10 mM EGTA, 50 μM BAPTA or 50 μM quin-2 for 20 min and then treated with 1 mM H_2_O_2 _for 2 h. In the presence of EGTA, BAPTA and quin-2, the proportions of dead cells in H_2_O_2_-treated cultures were 38%, 34% and 36%, respectively, indicating that Ca^2+ ^chelators reversed the effect of H_2_O_2 _(Fig. 5). When HSV-1-infected cells were treated with EGTA, BAPTA or quin-2 only, there were no changes in the proportion of dead cells (data not shown).

### Flow cytometric analysis of the H_2_O_2_-treated cells

**Figure 6:**
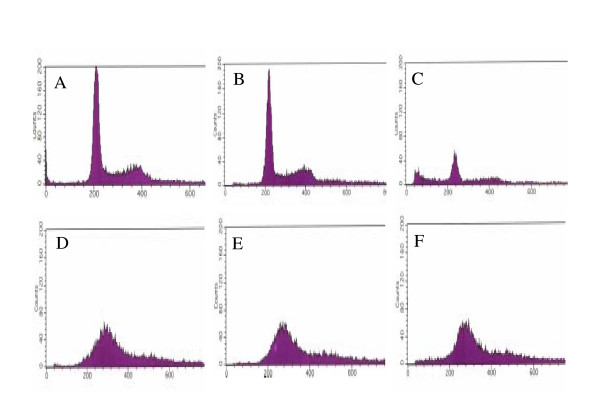
**Flow cytometric analysis of DNA fragmentation**. Untreated FI cells (A) and FI cells treated with 1 mM H_2_O_2 _for 2 h (B) or 24 h (C) were subjected to flow cytometric analysis. FI cells were infected with HSV-1 at an MOI of 2 PFU/cell and cultured for 20 h (D). HSV-1-infected cells were treated with 1 mM H_2_O_2 _from 18 to 20 h p.i. (E) or from 18 to 42 h p.i. (F). These infected cells were also subjected to flow cytometric analysis.

A number of studies have shown that H_2_O_2 _induced apoptosis with DNA fragmentation [8-11]. To clarify this issue, DNA was labeled by propidium iodide (PI) and subjected to flow cytometric analysis. In mock-infected cells treated with 1 mM H_2_O_2 _for 2 h, there were no apparent changes in the pattern of the cell cycle as compared with the untreated control (Fig. 6A and 6B). However, after treatment for 24 h, a sub-G1 peak appeared (Fig. 6C), indicating the induction of DNA fragmentation. When FI cells were infected with HSV-1 at an MOI of 2 PFU/cell and cultured for 18 h, the profile of DNA content was different from that of mock-infected cells. A broad peak was observed at the position of G_0_/G_1 _and the population of G_2_/M phase was decreased (Fig. 6D), indicating the disturbance of cell cycle due to HSV-1 infection. Even if infected cells were treated with 1 mM H_2_O_2 _for 2 h or 24 h, a specific sub-G1 peak was not demonstrated (Fig. 6E and 6F)

When HSV-1-infected cells were treated with 1 mM H_2_O_2 _from 18 to 20 h after infection and subjected to Hoechst staining and annexin V staining, increase of apoptotic cells was not demonstrated (data not shown)

### Electron microscopic observation

**Figure 7:**
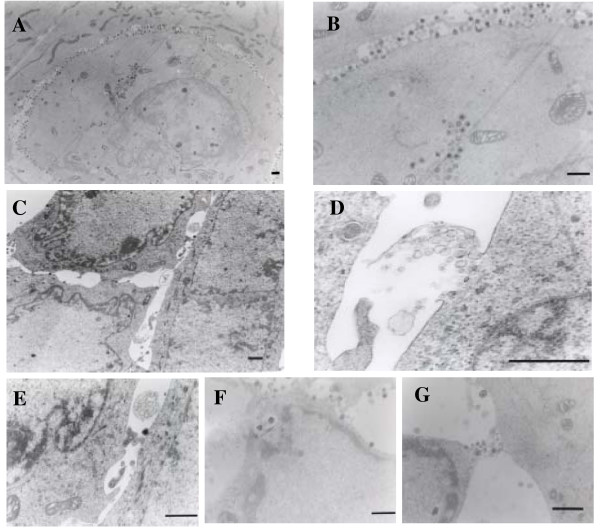
**Electron microscopic observation**. FI cells were infected with HSV-1 at an MOI of 2 PFU/cell and cultured for 20 h (A, B). The infected cells were also treated with 1 mM H_2_O_2 _for 18 to 20 h p.i. (C to G). To examine cell-to-cell interaction, cultures were fixed *in situ *and embedded in epoxy resin. Sections were cut parallel to the surface of the dishes. Bar, 1 μm

To gain further insight into the alterations caused by H_2_O_2_, electron microscopy was used. The cultures were fixed *in situ *and sections parallel to the dish surface were prepared. HSV-1-infected cells had large vesicular nuclei with dispersed chromatin. In the portion where cell-to-cell interaction was tight, a large number of viral particles were pooled in a narrow intercellular space (Fig. 7A and 7B). When HSV-1-infected cells were treated with 1 mM H_2_O_2 _from 18 to 20 h p.i., ruffling of the nuclear membrane and clustering of condensed chromatin at the nuclear periphery were observed, but the nuclear and cytoplasmic density was apparently unaltered. Cell shrinkage observed in apoptotic cells was not demonstrated. Generally, cell-to-cell junctions were enlarged, and as a consequence, viral particles pooled in the space were lost (Fig. 7C). Although the integrity of most of the plasma membrane was preserved, there were bubble-like structures that arose from the cell membrane (Fig. 7E). Occasionally, rapture of vacuoles containing organelles was observed on the cell surface (Fig. 7D). A focal defect of the plasma membrane was observed adjacent to transport vesicles containing viral particles at cell periphery (Fig. 7F and 7G).

## Discussion

We found that treatment with 1 mM H_2_O_2 _for 2 h significantly increased the amount of cell-free virus. If H_2_O_2 _could affect the step of virus release only, the increase of cell-free virus would be accompanied by the decrease of cell-associated virus, but the amount of cell-associated virus was not altered. This suggested that the total amount of infectious virus in the cultures was rather increased. Many factors such as cell proliferation and activity of protein and DNA synthesis will influence virus release and infectivity. It is possible that oxidative stress promotes the steps of transport and/or maturation of virus particles. Alternatively, H_2_O_2_-induced increase of [Ca^2+^]i may have an advantage of the infectivity of virions, because HSV-1 envelope was implicated to be sensitive to calcium depletion [21]. In any case, it is apparent that the proportion of cell-free virus in the cultures was markedly increased after treatment with H_2_O_2_. H_2_O_2 _must increase the release of HSV-1 at the final step of viral replication.

H_2_O_2 _exerts its effect through a second messenger, Ca^2+^, which may play a critical role in cellular events [8-12] and, probably, the process of HSV-1 replication. In the present study, there were two stages to the rise in [Ca^2+^]i ; an initial peak which appeared just after the addition of H_2_O_2_, followed by a secondary increase which persisted for some time. The removal of extracellular Ca^2+ ^by EGTA diminished the second rise in [Ca^2+^]i in response to H_2_O_2_, indicating that the secondary increase was due to Ca^2+ ^influx. The first peak was caused by the mobilization of Ca^2+ ^from intracellular stores [12,20] and both rises in [Ca^2+^]i were suppressed by the buffering agents BAPTA and quin-2 [22]. It is likely that H_2_O_2 _increases [Ca^2+^]i through the release of Ca^2+ ^from intracellular stores and Ca^2+ ^influx in HSV-1-infected cells. Since the buffering of [Ca^2+^]i by Ca^2+ ^chelators diminished the effect of H_2_O_2 _on the release of HSV-1, we concluded that the enhanced viral release following H_2_O_2 _treatment was ascribed to a Ca^2+ ^-mediated mechanism.

Oxygen radicals act as an inducer of apoptosis by elevating [Ca^2+^]i [9,11]. We found that a short-term treatment with H_2_O_2 _increased the number of dead cells in HSV-1-infected cultures and the effect was diminished in the presence of calcium chelators. However, a specific sub-G_1 _peak indicating apoptosis was not detected after H_2_O_2_treatment for 2 h by a flow cytometric analysis. Induction of apoptosis was not demonstrated by Hoechst staining and annexin V staining. Thus, the H_2_O_2_-induced cell death occurred in this situation was not apoptosis. The apoptosis of HSV-1-infected cells by H_2_O_2 _may be prevented the function of anti-apoptotic genes such as Us3, ICP27 and γ_1 _34.5 of HSV-1 [23-25].

The plasma membrane is the primary target of cell injury and the functional consequence of damage to this membrane is a lethal influx of extracellular Ca^2+ ^into the cells [26]. We also indicated that treatment of HSV-1-infected epithelial cells with ionomycin induced the increase of Ca^2+ ^influx, followed by cell death and the leakage of virus particles [20]. In the present study, H_2_O_2_-induced cell death was accompanied by the elevation of [Ca^2+^]i. Furthermore, with the use of an electron microscope, membrane protrusion, a bursting bubbles and a leakage of virus particles in H_2_O_2_-treated cells were observed. Thus, we concluded that the H_2_O_2_-induced cell death was characterized by a focal disintegration of the plasma membrane and partial loss of cytoplasmic contents, leading to the enhanced release of virus particles to the extracellular space. It should be also stated that the integrity of the nucleus and cytoplasmic density were preserved to produce progeny virus and the release of virus particles during the H_2_O_2_-induced cell death.

Another finding was that a number of cell-free viral particles were pooled at narrow cell junctions and were lost after treatment with H_2_O_2_, because of the enlargement of cell-to-cell junctions. As a function of a rise in [Ca^2+^]i, the cytoskeletal architecture and rigid intercellular connections are altered [27,28], which will result in the liberation of trapped viral particles from cell junctions. This must contribute to the increase in the amount of cell-free virus in HSV-1-infected cell cultures.

Oxygen radicals, such as H_2_O_2_, O_2_^•- ^and HO^•^, are highly reactive molecules with unpaired electrons that are generated in normal physiological processes such as aerobic metabolism or inflammation. PMNs generate both extracellular and intracellular oxygen radicals and the released oxygen radicals impair the host tissues [29,30]. The maximal H_2_O_2 _concentration was reported to be 0.3 mM after an activation of human PMNs [31]. Although 0.5 mM H_2_O_2 _increased cell-free virus (Fig. 1), we performed most experiments at H_2_O_2 _concentration of 1 mM. We speculate that a similar event would occur in vivo, because other PMN-derived oxygen radicals such as O_2_^•- ^and HO^• ^also exhibit cytotoxic effect [32]. In other systems to study the neuronal cell death and renal tubular cell injury by oxygen radicals, H_2_O_2 _was used at 1 mM [8,10]. Histological changes of skin vesicles due to HSV infection represent a combination of virally mediated cellular death and associated inflammatory response [33]. Oxygen radicals produced by inflammatory cells may promote the development of herpetic vesicular lesions by increasing the virus particles in the fluid. In mucosal lesions, more cell-free virus particles would be released from the ulcerative surface by the action of oxygen radicals and contribute to the spread of viral infection. Oxygen radicals also act as the mediators of anticancer agents [34,35]. This means that HSV-1 infection, irrespective of primary and recurrent infection, can be modified by antineoplasic agents, which may lead to the development of oral mucositis during antineoplastic chemotherapy [36]. From the aspect of exogenous oxygen radicals, H_2_O_2 _is used as a disinfectant, hemostatic or bleaching agent for colored tooth at a concentration of approximate 1 M. It can be a stimulator of viral release after a dilution to the level of mM in the oral cavity.

## Conclusion

Previously, we reported that a calcium ionophore, ionomycin, enhanced the release of HSV-1. Here, we indicated that treatment with H_2_O_2_disrupted cell-to-cell interactions, increased dead cells, and accelerated viral release through a Ca^2+^-mediated mechanism. H_2_O_2 _can be the candidate that elevates [Ca^2+^]i and promotes the release of HSV-1 in vivo.

## Methods

### Cell culture and virus

Oral squamous cell carcinoma FI cells [37] were used as an epithelial cell line throughout the experiments. FI cells were grown in Dulbecco's modified Eagle's medium containing 5% fetal bovine serum and supplemented with a penicillin-streptomycin antibiotic mixture. The stock of HSV-1 strain KOS was grown and infectivity was determined by plaque assay in Vero cells.

### Preparation of cell-free viral and cell-associated viral fractions

To measure the amounts of cell-free virus, FI cells were infected with HSV-1 at an MOI of 2 PFU/cell. Thereafter, the infected cells were cultured for 18 h and then treated with H_2_O_2_. The culture plates were centrifuged at 400 × g for 5 min and the supernatant was harvested as a cell-free fraction and stored at -80°C until use. An equal volume of medium was added to each culture plate. For the measurement of cell-associated virus in a culture, the cells were subjected to two cycles of freezing and thawing. They were then centrifuged and the supernatant was harvested as a cell-associated fraction and stored at -80°C. The viral titer in each fraction was measured by assaying the formation of plaques in Vero cell monolayers and means of three determinations were obtained. Results were compared to those for the untreated controls and a percentage value was calculated. Differences of means were analyzed with the unpaired *t*-test.

### Measurement of [Ca^2+^]i

[Ca^2+^]i was measured using the fluorescent Ca^2+ ^indicator fura-2, which was incorporated intracellularly as its acetoxymethyl ester (fura-2/AM; Calbiochem, Cambridge, MA, USA). Cells were grown on glass-based plastic dishes and incubated with 4 μM fura-2/AM in DMEM for 30 min at 37°C. Cells were then washed in modified Hank's solution (Sigma) containing 137 mM NaCl, 3.5 mM KCl, 0.44 mM KH_2_PO_4_, 25 mM NaHCO_3_, 0.33 mM Na_2_HPO_4 _and 0.5 mM CaCl_2 _for a further 20 min at room temperature. To deplete extracellular Ca^2+^, cells were treated with 10 mM EGTA (Calbiochem) for 10 min prior to the H_2_O_2 _treatment. For buffering [Ca^2+^]i, cells were pretreated with 50 μM of the acetoxymethyl ester of BAPTA (BAPTA/AM; Calbiochem) or 50 μM of the acetoxymethyl ester of quin-2 (quin-2/AM; Calbiochem) for 10 min. After the addition of H_2_O_2_, [Ca^2+^]i was measured in individually identified fura-2-loaded cells using alternating excitation wavelengths (340 and 380 nm) with an AQUACOSMOS ratio imaging application software (HAMAMATSU Photonics, Hamamatsu, Japan) and an inverted epifluorescence microscope (DIAPHOT 300, Nikon). In order to evaluate its ability to quantify [Ca^2+^]i, the instrument was tested on Ca^2+ ^buffer solutions (Molecular Probes) with known values of [Ca^2+^]i, using fura-2/AM [38]; 7 cells were monitored for each experiment.

### Trypan blue staining

Cell viability was determined by trypan blue dye exclusion analysis. Cells dissociated by the EDTA-trypsin solution were mixed with an equal volume of phosphate-buffered saline containing 0.24% trypan blue and observed with a microscope. We counted the numbers of stained and unstained cells. Results were compared to those for the untreated controls and a percentage value was calculated. Differences of means were analyzed with unpaired *t*-test.

### Flow cytometric analysis

FI cells were dissociated in the EDTA-trypsin solution. Isolated cells were added to ice-cold 70% ethanol and then incubated at -20°C for 4 h. Thereafter, cells were centrifuged and incubated with phosphate-citrate buffer for 30 min at room temperature. They were again centrifuged, incubated with 10 μg/ml PI and 10 μg/ml RNase A for 20 min at room temperature, and then analyzed with a Becton Dickinson FACSort (Becton Dickinson, San Jose, CA).

### Electron microscopy

Cells grown on plastic dishes were fixed in 2% glutaraldehyde (TAAB, Berkshire, England) for 2 h, washed with sodium cacodylate buffer and then postfixed in 1% osmium tetroxide (TAAB) for 2 h. Thereafter, cells were dehydrated in a graded series of ethanol and flat embedded in epoxy resin. Sections were cut parallel to the surface of the dishes. They were then stained with 4% uranyl acetate and 0.1% lead citrate (TAAB) and examined with a HITACHI H-7500 electron microscope.

## Competing interests

The author(s) declare that they have no competing interests.

## Authors' contributions

EA and YY conceived of the study, analyzed the results and wrote the manuscript. SI measured [Ca^2+^]i; TS performed flow cytometric analysis; YO carried out electron microscopic study. All authors read and approved the final manuscript.
